# Phosphatidylinositol (4,5) Bisphosphate Controls T Cell Activation by Regulating T Cell Rigidity and Organization

**DOI:** 10.1371/journal.pone.0027227

**Published:** 2011-11-11

**Authors:** Yi Sun, Radhika D. Dandekar, Yuntao S. Mao, Helen L. Yin, Christoph Wülfing

**Affiliations:** 1 Department of Immunology, UT Southwestern Medical Center, Dallas, Texas, United States of America; 2 Department of Physiology, UT Southwestern Medical Center, Dallas, Texas, United States of America; 3 Department of Cell Biology, UT Southwestern Medical Center, Dallas, Texas, United States of America; University Paris Sud, France

## Abstract

Here we investigate the role of Phosphatidylinositol (4,5) bisphosphate (PIP_2_) in the physiological activation of primary murine T cells by antigen presenting cells (APC) by addressing two principal challenges in PIP_2_ biology. First, PIP_2_ is a regulator of cytoskeletal dynamics and a substrate for second messenger generation. The relative importance of these two processes needs to be determined. Second, PIP_2_ is turned over by multiple biosynthetic and metabolizing enzymes. The joint effect of these enzymes on PIP_2_ distributions needs to be determined with resolution in time and space. We found that T cells express four isoforms of the principal PIP_2_-generating enzyme phosphatidylinositol 4-phosphate 5-kinase (PIP5K) with distinct spatial and temporal characteristics. In the context of a larger systems analysis of T cell signaling, these data identify the T cell/APC interface and the T cell distal pole as sites of differential PIP_2_ turnover. Overexpression of different PIP5K isoforms, as corroborated by knock down and PIP_2_ blockade, yielded an increase in PIP_2_ levels combined with isoform-specific changes in the spatiotemporal distributions of accessible PIP_2_. It rigidified the T cell, likely by impairing the inactivation of Ezrin Moesin Radixin, delayed and diminished the clustering of the T cell receptor at the cellular interface, reduced the efficiency of T cell proximal signaling and IL-2 secretion. These effects were consistently more severe for distal PIP5K isoforms. Thus spatially constrained cytoskeletal roles of PIP_2_ in the control of T cell rigidity and spatiotemporal organization dominate the effects of PIP_2_ on T cell activation.

## Introduction

Here we address roles of Phosphatidylinositol (4,5) bisphosphate (PIP_2_) in T cell activation. Physiological T cell activation occurs in the cellular interaction between a T cell and an antigen presenting cell (APC). T cells polarize upon APC contact as driven by the cytoskeleton [1], [2], [3], yielding a complex organization of T cell signaling in dynamic and diverse spatiotemporal patterns [4], [5], [6]. Prominent is the sustained accumulation of the T cell receptor (TCR) at the center of the T cell/APC interface [5], an accumulation pattern that can be associated with efficient T cell activation [6], [7]. A critical outcome of T cell activation is cytokine secretion, prominently that of the autocrine growth factor IL-2. PIP_2_ is a central substrate for second messenger generation and a well-established regulator of cytoskeletal dynamics in many cell types [8], [9]. Hydrolysis of PIP_2_ by phospholipase Cγ (PLCγ) yields diacylglycerol (DAG) and inositol 1,4,5 trisphosphate (IP_3_) [10], two signaling intermediates critical for the induction of T cell IL-2 secretion. In cytoskeletal regulation, PIP_2_ controls cytoskeleton-plasma membrane adhesion, the activity of actin severing proteins, and assembly of endocytic vesicles [9], [11], [12]. Ezrin Radixin Moesin (ERM) proteins are a critical mediator of PIP_2_ function in the regulation of cytoskeleton-plasma membrane adhesion, as binding of ERM to PIP_2_ in the plasma membrane activates them to strengthen the association of the plasma membrane with the underlying cortical actin cytoskeleton [13], [14]. A first general challenge in understanding the function of PIP_2_ in any cell type is to determine whether the role of PIP_2_ as a substrate for second messenger generation or cytoskeletal roles dominate the effects of PIP_2_ on cellular activation. In other words, we had to investigate whether changes in PIP_2_ levels primarily affected T cell activation through altered second messenger generation or through altered cytoskeletal dynamics.

PIP_2_ is turned over rapidly. The principal biosynthetic pathway of PIP_2_ involves phosphorylation of phosphatidylinositol 4-phosphate by the type I phosphatidylinositol 4-phosphate 5-kinases (PIP5K) [15]. There are three PIP5K isoforms, α, β, and γ The nomenclature for the α and β isoforms is switched between humans and mice. We use the more widely employed human nomenclature. The γ isoform has multiple splice variants. The predominant isoforms are PIP5K γ87 (also called γ635) and γ90 (γ661) with the isoforms being denoted by their molecular weight (87 or 90 kDa) or the number of amino acids (635 or 661). PIP_2_ is metabolized through hydrolysis by PLCγ or phosphorylation by phosphatidylinositol 3-kinase (PI3K). Additionally, PIP_2_ synthesized locally will be dissipated by diffusion [16] unless captured by scaffolding molecules [17]. PIP_2_ is also dephosphorylated by phosphatidylinositol phosphatases [18]. A second key challenge in understanding roles of PIP_2_ in any cell type is to gain comprehensive insight into how PIP_2_ turnover is regulated by this complex group of pathways. As proteins that generate, metabolize, or function as effectors of PIP_2_ often display distinct subcellular localization, the complex PIP_2_ turnover needs to be analyzed with resolution in time and space. In other words, we had to determine where and when PIP_2_ was synthesized and degraded during T cell activation. As we have already characterized spatiotemporal distributions of PIP_2_ hydrolysis by PLCγ and of PIP_2_ phosphorylation by PIP3K at the T cell/APC interface as part of a larger systems analysis [6], we focus here on the spatiotemporal characteristics of PIP_2_ generation. Once it was better understood when and where PIP_2_ was turned over, it was important to elucidate how the spatiotemporal constraints of PIP_2_ turnover govern PIP_2_ function. In other words, we had to investigate how manipulation of PIP_2_ localization affected roles of PIP_2_ in T cell activation.

During T cell activation, PIP_2_ is rapidly synthesized and hydrolyzed [19], [20], even though the spatiotemporal characteristics of PIP_2_ synthesis are unknown. Cytoskeletal roles of PIP_2_ in T cell activation are also unresolved. They are likely prominent as PIP_2_ regulates polarization in many related cell types. In macrophages, PIP_2_ stabilizes actin at the phagocytic cup [21] and PIP5K γ and α are critical in actin-dependent binding and internalization of particles [22]. In neutrophils, PIP5K β and γ90 are critical for the turnover of the uropod, a posterior cell extension involved in adhesion in particular during the extravasation of blood cells from the vasculature [23], [24], [25]. In lymphocyte responses to chemokines, hydrolysis of PIP_2_ is critical in increasing cellular flexibility during extravasation as associated with inhibition of ERM activity [26].

Here we address the two general challenges of PIP_2_ biology, the balance between roles of PIP_2_ as a substrate for second messenger generation versus cytoskeletal roles and the complexity of PIP_2_ turnover with resolution in time and space with its functional consequences, in primary T cells. We found that PIP_2_ synthesis occurs with distinct spatiotemporal characteristics, as four PIP5K isoforms (αβγ87, and γ90) displayed distinct dynamic localization during T cell activation. Overexpression in particular of the distal PIP5K isoforms, β and γ90, yielded a general, yet modest increase in PIP_2_ levels combined with distinct isoform-specific changes in the spatiotemporal distributions of accessible PIP_2_. Such overexpression, as corroborated by knock down and PIP_2_ blockade, established that PIP_2_ primarily controls T cell activation using spatially constrained cytoskeletal means, by regulating T cell rigidity and the spatiotemporal organization of T cell signaling with an emphasis on the T cell distal pole.

## Results

### Different PIP5Ks are enriched in distinct locations during T cell activation

As a critical foundation for the understanding of the complexity of PIP_2_ turnover in T cells, we determined the spatiotemporal features of PIP_2_ synthesis. Primary T cells expressed all three PIP5K isoforms, α, β, and γ with both the γ87 and γ90 splice variants of the γ isoform (Fig. S1A), as determined by real time PCR. To elucidate the spatiotemporal distributions of PIP5K isoforms during the activation of primary T cells, we used in vitro primed primary T cells from 5C.C7 TCR transgenic mice. The 5C.C7 TCR recognizes peptide 83–102 of moth cytochrome C (MCC) as presented by the MHC II allele I-E^k^. We retrovirally transduced T cells to express GFP-tagged PIP5K isoforms. We activated PIP5K expressing T cells with CH27 B cell lymphoma APC incubated with a high concentration (10 µM) of the MCC agonist peptide. These were the default T cell activation conditions for this study.

**Figure 1:**
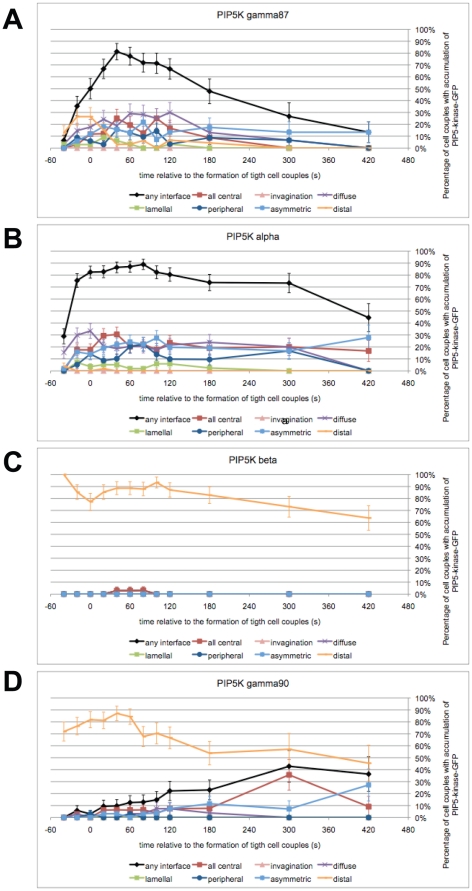
Different PIP5K isoforms display distinct spatiotemporal patterns. 5C.C7 T cells were transduced to express GFP-tagged PIP5K isoforms and activated with CH27 APCs and 10 µM MCC agonist peptide. The graphs show the percentage of cell couples with standard errors that displayed accumulation of PIP5K γ87-GFP (A), α-GFP (B), β-GFP (C), and γ90-GFP (D) with the indicated patterns [6] relative to tight cell coupling. Representative images are given in Fig. S1B–E. Representative movies have been published (γ87 [6]) or are given as Movies S1, S2, S3. 34–59 cell couples were analyzed per condition.

PIP5K γ87 was rapidly recruited to the T cell/APC interface such that 1 min after interface formation 74±8% of cell couples displayed PIP5K γ87 interface accumulation (Figs. 1A, S1B). PIP5K γ87 accumulation was transient such that 5 min after tight cell coupling only 27±11% cell couples with interface accumulation remained (Fig. 1A). Interface accumulation of PIP5K α also occurred preferentially at the cellular interface, however, in a more sustained fashion (Figs. 1B, S1C). In contrast, PIP5K β accumulated almost exclusively at the distal pole (Figs. 1C, S1D). Within the first two minutes after cell coupling the γ90 isoform preferentially accumulated at the distal pole (Figs. 1D, S1E). Subsequently however, a substantial portion of PIP5K γ90 moved to the T cell/APC interface reaching about 40% cell couples with interface accumulation ≥5 min after tight cell coupling (Fig. 1D). Interface accumulation of PIP5K γ87 without characterization of its spatiotemporal features has been described before [27].

The determination of the differential localization of the four PIP5K isoforms is a central contribution to the understanding of the complex regulation of PIP_2_ turnover in time and space, in particular in the context of our wider system analysis of T cell signaling [6], as discussed below. It also allowed us to manipulate PIP_2_ with spatial definition by overexpressing distinct PIP5K isoforms. For such studies we focused on the two distal PIP5K isoforms, γ90 and β, as it of interest to understand how regulation of PIP_2_ levels at the distal pole, away from the location of T cell signaling at the interface, would impact T cell activation.

### PIP5K overexpression yields increased PIP_2_ levels and isoform-specific changes in the patterns of accessible PIP_2_

**Figure 2:**
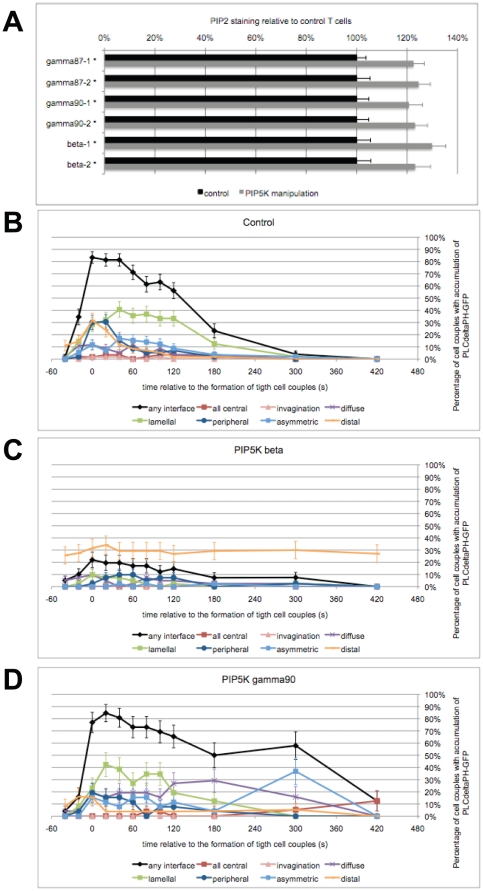
PIP5K overexpression alters PIP_2_ levels and localization. **A**. PIP_2_ levels were determined by immunofluorescence staining of 5C.C7 T cells transduced with different GFP-tagged PIP5K isoforms as indicated. PIP_2_ levels of T cells with PIP5K overexpression (grey bars) and control (black bars) are normalized to control and are given with standard errors for two independent experiments each. An asterisks indicates statistical significance versus control with p<0.005. 100–200 cells were analyzed per experiment. A representative PIP_2_ staining experiment is shown as Fig. S2D. **B**. 5C.C7 T cells were transduced to express PLCδ PH-GFP and activated with CH27 APCs and 10 µM MCC agonist peptide. The graph shows the percentage of cell couples with standard errors that displayed accumulation of PLCδ PH-GFP with the indicated patterns [6] relative to tight cell coupling. 60 cell couples were analyzed. **C, D**. Similar to B, the panels show patterning data for 5C.C7 T cells expressing PLCδ PH-GFP with concurrent overexpression of PIP5K β (C) or γ90 (D). 41, 26 cell couples were analyzed per condition. Representative images for panels B–D are given in Fig. S2E–G. Representative movies have been published (γ87 [6]) or are given as Movies S4, S5, S6.

To address roles of PIP_2_ in T cell activation under consideration of PIP_2_ localization, we used primarily PIP5K overexpression (Fig. S2A–C), with knockdown and PIP_2_ blockade for corroboration. PIP5K overexpression modestly but significantly (p<0.005) increased cellular PIP_2_ levels by ∼20% (Figs. 2A, S2D), as determined by immunostaining for PIP_2_. These data suggest that PIP_2_ levels were tightly controlled in T cells, similar to other cell types where PIP5K overexpression or deletion often affects PIP_2_ levels only modestly [22], [28], [29], [30].

To assess consequences of PIP5K overexpression on PIP_2_ localization, we used the well-established PIP_2_-binding PLCδ PH domain. As previously described [6], upon tight cell coupling 83±5% of cell couples showed PLCδ PH-GFP at the T cell/APC interface (Figs. 2B, S2E). Accumulation was transient, disappearing entirely at 7 min after tight cell coupling (Fig. 2B). Less than 1/3^rd^ of the cell couples also displayed transient distal accumulation of PLCδ PH-GFP within the first minute after tight cell coupling (Fig. 2B).

Upon overexpression of PIP5K β (Fig. 2C, S2F), interface accumulation of PLCδ PH-GFP was almost completely lost, not exceeding 25% of cell couples with such accumulation at any time (p<0.001 versus control between time points - 20 and 120 s). Instead, consistent accumulation of PLCδ PH-GFP at the distal pole was observed in about 1/3^rd^ of the cell couples at all time points (p<0.005 versus control at all time points ≥1 min)(Fig. 2C). This phenotype is consistent with the exclusive distal accumulation of PIP5K β itself (Fig. 1C). Overexpression of PIP5K γ90 yielded significantly (p<0.01) increased accumulation of PLCδ PH-GFP at the T cell/APC interface ≥3 min after its formation with as many as >50% of the cell couples showing such accumulation (Fig. 2D, S2G). This enhancement is consistent with translocation of PIP5K γ90 to the interface during this time (Fig. 1D). Interestingly, the early distal presence of PIP5K γ90 did not trigger distal accumulation of PLCδ PH-GFP, consistent with a lack of activators of PIP5K γ90 or rapid turnover of PIP_2_ at the distal pole at that time. For corroboration we used overexpression of the interface-localized PIP5K γ87 isoform. Such overexpression yielded a comparable increase in cellular PIP_2_ levels without substantial changes in PIP_2_ localization (Figs. 2A, S2H, S2I). We also reduced PIP_2_ generation and access with knockdown of the PIP5K γ isoforms (Fig. S2J) and PIP_2_ blockade with the PLCδ PH domain as a protein transduction reagent (Fig. S2K).

In summary, PIP5K overexpression yielded a modest but significant (p<0.005) increase in PIP_2_ levels together with isoform-specific changes in PIP_2_ localization. Isoform-specific effects of PIP5K overexpression thus will identify location-dependent roles of PIP_2_. Additional roles of PIP5K isoforms, such as in scaffolding as recently described for PIP5K α [31], cannot be ruled out.

### PIP5K overexpression impairs IL-2 secretion and proximal T cell signaling

**Figure 3:**
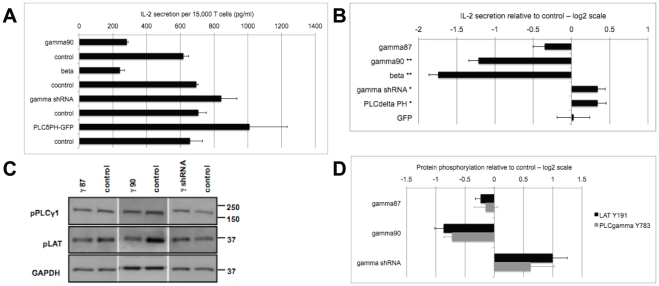
Manipulation of PIP5K expression affects IL-2 secretion and proximal signal transduction. **A, B**. 5C.C7 T cells were activated by CH27 APCs and 10 µM MCC agonist peptide for 16 h upon manipulation of PIP5K expression and PIP_2_ blockade as indicated. Cell culture supernatants were analyzed for IL-2 by ELISA. A representative IL-2 ELISA is given in (A). Changes in IL-2 secretion upon manipulation of PIP5K expression and PIP_2_ blockade relative to non-transduced T cells are given with standard errors in (B) on a logarithmic scale to comparably display reduction and enhancement. ‘GFP’ indicates retroviral expression of GFP as a control. One/two asterisks indicate significance versus non-transduced control with p<0.05/0.005, respectively. Data from 3–6 independent experiments are given. When the agonist peptide concentration during T cell activation was reduced to a limiting concentration, 0.1 µM, knockdown of PIP5K γ still did not result in impaired IL-2 secretion (Fig. S3). **C, D**. 5C.C7 T cells were activated by CH27 APCs and 10 µM MCC agonist peptide for 2 min upon manipulation of PIP5K expression as indicated. T cell/APC extracts were blotted for LAT Y191 and PLCγ Y783. A representative blot is given in (C). Changes in LAT and PLCγ phosphorylation upon manipulation of PIP5K expression relative to non-transduced T cells are given with standard errors in (D) on a logarithmic scale to comparably display reduction and enhancement. Data from 3–4 independent experiments are given.

IL-2 secretion is a key outcome of T cell activation. We therefore determined its dependence on PIP_2_. Overexpression of the distal PIP5K isoforms γ90 or β reduced IL-2 secretion to 44±4% and 30±3% of IL-2 secretion of control T cells, respectively (Fig. 3A, B)(p<0.005). In contrast, knockdown of PIP5K γ and 5 µM tatPLCδ PH modestly but significantly (p<0.05) enhanced IL-2 secretion by 26±9% (18±2% in a second set of experiments, Fig. S3) and 29±10%, respectively. Corroborating these data, complete loss of PIP5K γ90 leads to an increase in IL-2 secretion by about 40% [27]. The comparatively modest size of this enhancement is discussed below. Changes in IL-2 secretion upon overexpression of the interface-associated PIP5K isoform γ87 were not significant (Fig. 3B). These data are significant, first, as they establish PIP_2_, in particular distal PIP_2_, as a substantial regulator of T cell activation. Second, they are critical in understanding a potential role of PIP_2_ as second messenger precursor. If PIP_2_ as a substrate for PLCγ was central for mediating effects of PIP_2_ turnover on T cell activation, mass action dictates that increased PIP_2_ generation should yield increased second messenger levels and thus increased amounts of IL-2. As our findings run contrary to this expectation, roles of PIP_2_ as a second messenger substrate were surprisingly minor.

To assess whether PIP_2_ could function within minutes of T cell coupling to APCs, the time of drastic cytoskeletal rearrangements, of maximal biochemical signaling activity, and of the translocation of transcription factors to the nucleus [6], we addressed phosphorylation of linker of activated T cells (LAT) and phospholipase Cγ (PLCγ). Due to limiting cell numbers, the determination of tyrosine phosphorylation of LAT (Y191) and PLCγ (Y783) in T cell/APC extracts was restricted to overexpression of PIP5K γ90 and PIP5K γ87 as a non-distal control. LAT and PLCγ phosphorylation were significantly decreased (p<0.05) upon overexpression of PIP5K γ90 by 46±6% and 39±6% (Fig. 3C, D). In contrast, LAT phosphorylation was enhanced by 110±40% upon knockdown of PIP5K γ (p<0.05). Changes upon overexpression of PIP5K γ87 were not significant (Fig. 3D). Expression of total LAT and PLCγ has previously been shown to not vary during early primary T cell activation [32]. Importantly, effects of PIP_2_ manipulation on proximal signaling and IL-2 secretion were extensively matched, consistent with the suggestion that reduced efficiency of proximal T cell signaling was a key contributor to decreased IL-2 secretion.

### PIP_2_ regulates T cell spreading and uropod retraction upon APC contact

**Figure 4:**
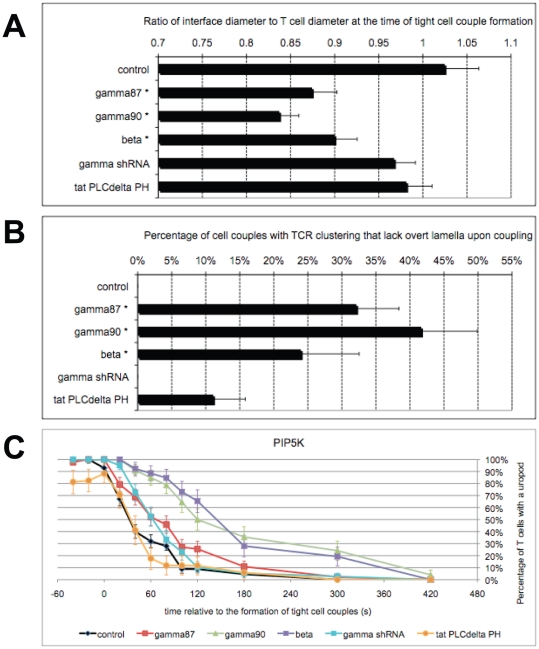
PIP5K overexpression rigidifies T cells. **A**. 5C.C7 T cells were activated with CH27 APCs and 10 µM MCC agonist peptide. The diameter of the T cell/APC interface at the time of tight cell coupling is given with standard errors relative to that of the T cell body upon manipulation of PIP5K expression as indicated. An asterisk indicates statistical significance with p<0.01 relative to control. 28–54 cell couples were analyzed per condition. **B**. 5C.C7 T cells were transduced to express TCRζ-GFP and were activated with CH27 APCs and 10 µM MCC agonist peptide. For all cell couples with persistent interface accumulation of TCRζ-GFP it was determined whether preceding cell coupling occurred with (e.g. Figs. S1C, S2E, and S6A) or without (Fig. S4A, Movie S7) the formation of a visible lamellum. Of all T cell/APC couples with persistent TCRζ-GFP interface accumulation, the percentage of cell couples without visible lamellum upon cell coupling is given with standard errors upon manipulation of PIP5K expression, as indicated. An asterisk indicates statistical significance with p<0.001 relative to control. 25–56 cell couples were analyzed per condition. **C**. 5C.C7 T cells were activated with CH27 APCs and 10 µM MCC agonist peptide. The percentage of cell couples with a visible uropod is given with standard errors relative to tight cell coupling upon manipulation of PIP5K expression and PIP_2_ blockade, as indicated. 17–68 cell couples were analyzed per condition.

As the data on IL-2 secretion suggest that changes in the amounts and/or localization of PIP_2_ do not substantially affect T cell activation through a role of PIP_2_ as a second messenger substrate, we next addressed cytoskeletal roles of PIP_2_. We started with the control of cellular rigidity, as this is a common function of PIP_2_ [15]. Overexpression of PIP5K β, γ87, or γ90 yielded smaller initial T cell/APC interface diameters. To account for variable T cell size, we determined the interface diameter relative to the diameter of the T cell body. In control T cells at the time of tight cell coupling, the interface diameter was 1.02±0.03 times that of the T cell body (Fig. 4A). It was significantly reduced to 0.84±0.02 to 0.90±0.03 times the diameter of the cell body upon overexpression of each of the PIP5K isoforms (p<0.01 versus control, no significant differences between PIP5K isoforms), indicative of increased T cell rigidity.

Impaired T cell spreading became even more evident in experiments to assess TCR clustering at the T cell/APC interface. In these experiments the majority of productive T cell/APC couples displayed accumulation of the TCR at the center of the T cell/APC interface, as discussed in detail below. In control T cells, such accumulation was invariably preceded by spreading of a clearly visible wide lamellum against the APC [6]. However, upon overexpression of PIP5K isoforms, we observed TCR clustering without a visible lamellum and the formation of a wide T cell/APC interface (Fig. S4A) in a substantial portion of the cell couples. As we could not determine the precise time of cell coupling and its associated interface diameter in these cell couples any more, we used an alternate measurement: When we analyzed all T cell/APC couples with eventual interface accumulation of the TCR, upon overexpression of PIP5K β, γ87, or γ90, 24±9% to 41±8% of the cell couples did not display a lamellum upon cell coupling (p<0.001 versus control, no significant differences between PIP5K isoforms)(Figs. 4B, S4A), indicative of most severely increased T cell rigidity.

As a second cell shape change upon APC coupling, the T cell retracts its uropod. Overexpression of PIP5K β or γ90 specifically delayed uropod retraction (Figs. 4C, S4B), another indication of increased T cell rigidity, this time focused on the T cell distal pole. In control cells at 2 min after tight cell coupling, only 9±3% of cell couples still displayed a visible uropod. This percentage was significantly (p<0.001) increased upon overexpression of PIP5K β or γ90 to 65±9% and 50±9%.

In summary, overexpression of PIP5K isoforms substantially interfered with T cell shape changes upon APC contact, both at the interface and at the distal pole, indicative of increased T cell rigidity. PIP5K γ knockdown or PIP_2_ blockade altered neither the interface diameter at T cell/APC coupling nor uropod retraction. Effects of PIP5K gamma knockdown of PIP_2_ levels were limited (Fig. S2J) and blockade of PIP_2_ by the PLCδ PH domain was partial (Fig. S2K), providing potential technical explanations for the more limited nature of these effects. A possible biological explanation is that T cell flexibility might have already been maximized upon T cell activation with a strong stimulus, thus preventing further enhancement upon interference with PIP_2_ generation or access. Our data on T cell rigidity are corroborated by data on actin spreading to the edge of the T cell/APC interface (Fig. S4C–L). Importantly, effects of PIP_2_ on T cell rigidity and IL-2 matched. Increased T cell rigidity should interfere with T cell activation by impairing tight APC coupling and thus extensive receptor ligand engagement. Accordingly, increased PIP_2_ generation in parallel made T cells more rigid and interfered with IL-2 secretion. Interestingly, local features of PIP_2_ turnover contributed substantially, as effects of overexpression of distal PIP5K isoforms were more dramatic both in the control of T cell rigidity, involving uropod retraction, and IL-2 secretion.

### PIP5K overexpression impairs ERM protein dephosphorylation upon T cell activation

**Figure 5:**
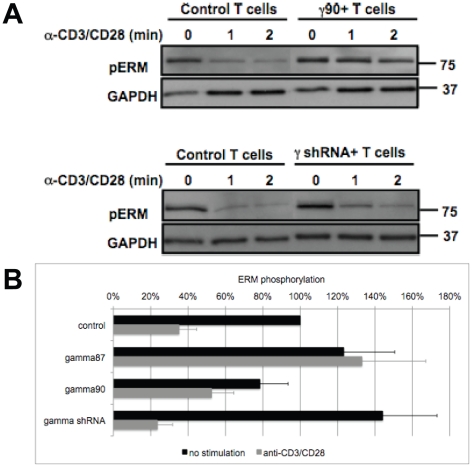
PIP5K overexpression interferes with ERM dephosphorylation. **A, B**. 5C.C7 T cells were activated with α-CD3 and α-CD28 antibodies for 1 or 2 min upon manipulation of PIP5K expression as indicated. T cell extracts were blotted for phospho-Ezrin T567/Radixin T564/Moesin T558. Representative blots are given in (A). Phospho-ERM levels after two minutes of T cell activation are given with standard errors relative to those of T cell extracts from non-stimulated, non-transduced T cells in (B). Modest differences in ERM phosphorylation prior to T cell stimulation were not significant. Data from 3–10 independent experiments are given.

T cell rigidification could be caused by altered ERM protein function, as ERM proteins link the plasma membrane to the cortical actin cytoskeleton in a PIP_2_-dependent fashion. ERM protein activity requires threonine phosphorylation at residues T567 of Ezrin and T558 of Moesin [33], [34], [35], [36]. We therefore determined ERM threonine phosphorylation during T cell activation. TCR engagement was provided by α-CD3 plus α-CD28 antibodies, as the determination of T cell ERM phosphorylation in T cell/APC couples, where both cell types contain ERM proteins, is challenging. Due to limiting cell numbers, these experiments were restricted to overexpression of PIP5K γ90 and PIP5K γ87 as a non-distal control. In non-transduced T cells, ERM phosphorylation dropped significantly (p<0.001) to 35±9% of the level of non-stimulated T cells within 2 min of tight cell coupling (Fig. 5). This reduction is indicative of the increased T cell flexibility required to execute the T cell shape changes associated with T cell activation. However, upon overexpression of PIP5K γ90, ERM phosphorylation dropped significantly less (γ90)(p≤0.01) to only 66±5% of the level of non-stimulated T cells (Fig. 5). Overall ERM expression was not altered by PIP5K overexpression (Fig. S5A). We thus suggest as a mechanism of PIP5K-dependent T cell rigidification that elevated PIP_2_ generation inhibits ERM inactivation upon T cell activation. Upon knockdown of PIP5K γ, T cell stimulation triggered slightly enhanced reduction in ERM phosphorylation to 19±7% of the prestimulation levels (Fig. 5, not significantly different from control however) as consistent with unimpaired T cell flexibility. A reduced frequency of T cell coupling and impaired F-actin polymerization could be ruled out as alternate mechanisms of PIP_2_ action (Fig. S5B, C).

### The spatiotemporal organization of T cell signaling depends on PIP_2_

**Figure 6:**
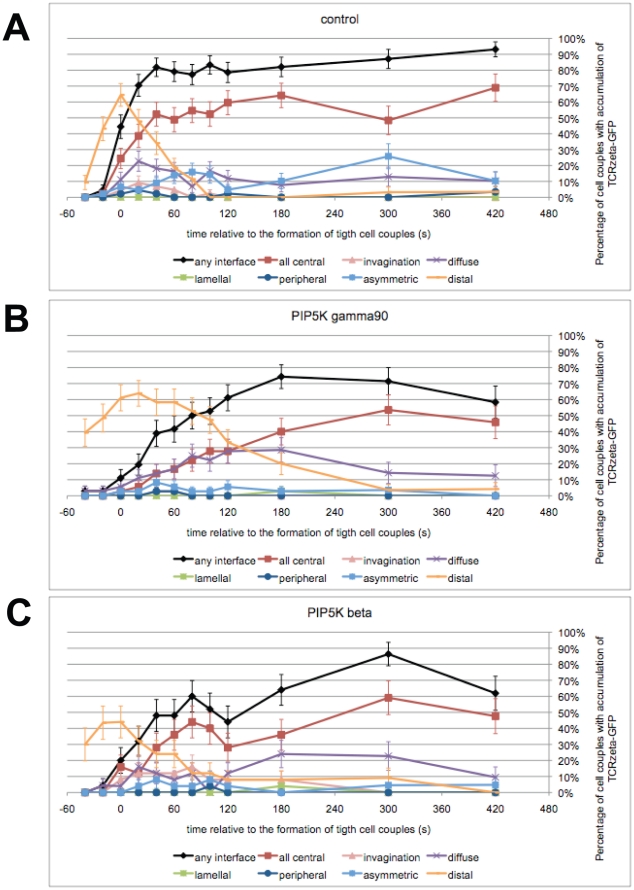
PIP5K overexpression interferes with accumulation of the TCR at the center of the T cell/APC interface. **A**. 5C.C7 T cells were transduced to express TCRζ-GFP and activated with CH27 APCs and 10 µM MCC agonist peptide. The graph shows the percentage of cell couples with standard errors that displayed accumulation of TCRζ-GFP with the indicated patterns [6] relative to tight cell coupling. 45 cell couples were analyzed. **B, C**. Patterning data for 5C.C7 T cells expressing TCRζ-GFP upon concurrent overexpression of PIP5K γ90 (B) or β (C) are displayed as in (A). 36, 25 cell couples were analyzed per condition. Representative images for all three panels are given in Fig. S6A–C. Representative movies are given as Movies S12, S13, S14.

To understand how T cell rigidity may be linked to T cell signaling, we addressed effects of PIP_2_ on TCR clustering. TCR clustering as a representative element of the system wide spatiotemporal organization of T cell signaling [6], is dependent of cytoskeletal dynamics [37], [38], and is thus likely to be influenced by ERM-regulated cytoskeleton plasma membrane interactions. Upon tight cell coupling, the TCR is recruited to the T cell/APC interface with a preference for the interface center. In the activation of 5C.C7 T cells by APCs such central clustering is related to the efficiency of T cell signaling [6], [7]. Moreover, TCR clustering has a strong distal component as in about half of the cell couples the TCR is recruited transiently to the T cell distal pole [6](Figs. 6A, S6A). Rapid release of the TCR from the distal pole is associated with increasing interface accumulation.

Overexpression of PIP5K β and γ90 interfered with TCR accumulation at the T cell/APC interface within the first minute of tight cell coupling (Figs. 6B, C, S6B, C). For example upon overexpression of PIP5K γ90, interface accumulation of the TCR in any pattern and in a central pattern at 1 min was reduced from 79±6% and 49±8% of control T cell/APC couples with such accumulation to 42±8% and 17±6%, respectively (p<0.005)(Fig. 6B). Delayed interface accumulation was accompanied by delayed TCR release from the distal pole. For example at 2 min no distal pole accumulation was observed in control T cell/APC couples any more, while in cell couples with T cell overexpression of PIP5K γ90 or β 33±8% and 8±5% of cell couples, respectively, still displayed distal accumulation of the TCR (p≤0.01)(Fig. 6B, C). The phenotype upon PIP5K β overexpression was most complex, as both the inducible recruitment of the TCR to the distal pole and the subsequent release were defective (Fig. 6C). Intriguingly, the severity of the defects in the early spatiotemporal organization of T cell signaling upon overexpression of the different PIP5K isoforms matched the degree of impairment of IL-2 secretion, in that distal PIP5K isoforms were most effective. Effects of overexpression of PIP5K γ87 (Fig. S6D, E), PIP5K γ knockdown (Fig. S6F, G), and PIP_2_ blockade (Fig. S6H, I) were less severe to not significant, again consistent with data on IL-2 secretion.

The TCR localization experiments tie our data together into a consistent scenario of how cytoskeletal roles of PIP_2_ control T cell activation: Increased rigidity of the T cell upon PIP5K overexpression (Figs. 4, S4) could trap the TCR, in particular at the distal pole (Fig. 6), thus delaying and impairing the formation of a TCR-anchored signaling complex at the center of the T cell/APC interface (Fig. 6). A smaller interface would also limit receptor engagement. Less central TCR clustering and receptor engagement is linked to less efficient proximal signaling in 5C.C7 T cell/APC couples [6], [7], as also seen here (Fig. 3C, D), and thus to less IL-2 secretion (Fig. 3A, B).

## Discussion

Here we have addressed two principal challenges in PIP_2_ biology in the activation of primary T cells. First, we found that changes in PIP_2_ levels affect T cell activation primarily through regulation of T cell rigidity and spatiotemporal organization, not through the role of PIP_2_ as a substrate in second messenger generation. Increased amounts of PIP_2_ did not yield increased IL-2 secretion, as to be expected for a central role of PIP_2_ as the substrate of PLCγ, but they resulted in substantially less IL-2 secretion. This decrease matched a series of data generated here, as summarized in Table S1. Increased PIP_2_ yielded a more rigid T cell, a T cell with delayed and impaired clustering of the TCR at the center of the T cell/APC interface, and impaired proximal T cell signaling. Importantly, these observations have previously been linked using the same experimental system. In the activation of 5C.C7 T cells by APC plus peptide, TCR clustering is dependent on intact actin dynamics that also regulate cell shape [37], [38] and central TCR clustering is related to efficient proximal signaling [6], [7]. In addition, the effects of altered PIP_2_ generation were comparably spatially constrained. Increased generation of PIP_2_ at the T cell distal pole as opposed to at the T cell/APC interface consistently had the strongest effects. While all PIP5K isoforms controlled T cell rigidity at the interface, consistent with their shared role in regulating cellular PIP_2_ levels, only the distal isoforms also rigidified the distal pole. Initial recruitment of the TCR to distal pole, its release and its central accumulation were all more dependent on the distal PIP5K isoforms, as were proximal signaling and IL-2 secretion. The shared spatial constraint further strengthens the functional connections between T cell rigidity, organization, and function. Significantly, it links spatial features of PIP_2_ turnover to those of PIP_2_ function. Our data thus establish that PIP_2_ regulates T cell activation primarily through spatially constrained control of T cell rigidity and spatiotemporal organization, thus forming a first critical foundation for future more detailed work on roles of PIP_2_ in T cell activation.

As a second general challenge, we have determined the spatiotemporal features of PIP_2_ synthesis as a critical part of a more comprehensive understanding of PIP_2_ turnover as resolved in time and space. Different PIP5K isoforms were enriched at the T cell/APC interface or the T cell distal pole with distinct local preferences and dynamics. As part of a larger analysis of the spatiotemporal organization of T cell signaling [6], the following picture emerges: The T cell/APC interface likely is the site of most intense PIP_2_ turnover. Not only are the two most abundant PIP5K isoforms (γ87 and α) enriched there, but also two key enzymes in PIP_2_ metabolism, PLCγ and PI3K. Based on the observed distributions of free PIP_2_, PIP_2_ synthesis dominated during the first minute of cell coupling, PIP_2_ metabolism thereafter. The distal pole in contrast is likely a site of slower PIP_2_ turnover. It displayed enrichment of only the less abundant PIP5K isoforms and none of PIP_2_ metabolizing enzymes. Nevertheless, initial PIP_2_ metabolism still seems active, as accumulation of accessible PIP_2_ after T cell/APC coupling was only transient (Fig. 2B). However at later time points, overexpression of the distal PIP5K β isoform yielded stable accumulation of free PIP_2_ at the distal pole (Fig. 2C), indicative of slowed PIP_2_ turnover and something not seen at all at the T cell/APC interface upon overexpression of interface-associated PIP5K isoforms. Slower turnover of distal PIP_2_ at later time points is consistent with the stable accumulation of the PIP_2_-binding ERM at the distal pole, as opposed to no or only transient accumulation at the T cell/APC interface [35]. This initial characterization of PIP_2_ turnover as resolved in time and space forms a second critical foundation for future more detailed work on roles of PIP_2_ in T cell activation.

## Materials and Methods

### Ethics Statement

All mouse studies have been approved by the UT Southwestern Medical Center Institutional Animal Care and Use Committee under protocol 2010-0224 and were executed in accordance with the USDA Animal Welfare Act.

### Cells and reagents

In vitro primed, primary T cells from 5C.C7 TCR transgenic mice were generated as described [37]. CH27 cells were used as APCs [37]. PIP5K-GFP fusion proteins were generated as fusions of GFP to the N-terminus of PIP5K. Actin-GFP, TCRζ-GFP, PLCδ PH-GFP have been described [6]. Retroviral transduction was performed as described [37]. T cells that retrovirally express fluorescent sensor proteins were FACS sorted for a consistent and low expression level. Through quantitative immnuoblotting with anti-GFP antibodies of cell extracts from the sorted T cells against dose responses of pure GFP, this sensor expression level was determined to be 5 µM [6]. For shRNA-mediated knockdown of PIP5K γ, a RNA polymerase II-driven cassette for parallel expression of an shRNA hairpin and a marker protein [39] was cloned into the MMLV-based retroviral vector. For retroviral expression of PIP5K together with imaging sensors from the same mRNA, sensor translation was initiated on an EMCV-based internal ribosomal entry site. The following antibodies were used: antibodies against phospho-LAT Y191, phospho-PLCγ Y783, Ezrin/Radixin/Moesin, phospho-Ezrin T567/Radixin T564/Moesin T558 (Cell Signaling, Danvers, MA), PIP5K γ (Epitomics, Burlingame, CA) antibodies against CD3 (2C11) and CD28 (BD Pharmingen), and polyclonal rabbit anti-PIP5K γ90 [40] and mouse anti-PIP_2_ sera (a kind gift from K. Fukami, U. Tokyo). Alexa Fluor 594 phalloidin was from Molecular Probes (Eugene, OR). A protein transduction version of the PLCδ PH domain was generated, purified under native conditions from E. coli by immobilized metal affinity chromatography, and applied to T cells in strict analogy to [41].

### Image acquisition and image analysis

Image acquisition and analysis were performed as described in great detail [6]. Briefly, T cell-APC interactions were imaged at 37°C. Every 20 seconds, 1 DIC and 21 fluorescence images that spanned 20 µm in the z-plane at 1 µm intervals were acquired. The acquisition and analysis software was Metamorph (Molecular Devices). The formation of a tight cell couple, time 0 in our analysis, was defined as either the first time point with a fully spread T cell-APC interface or 40 s after first membrane contact, whichever occurred first. A region of sensor accumulation was defined by an average fluorescence intensity of >135% of the background cellular fluorescence. To classify spatial accumulation features, six mutually exclusive interface patterns were used: central, invagination, diffuse, lamellal, asymmetric and peripheral, as defined by strict geometrical constraints (Table 2 and actin data in Figure S12 in [6]). Distal accumulation was scored independently. A T cell was scored to have a uropod as long as an inversion of curvature of the plasma membrane could be detected at the distal pole in the DIC images. To ensure the reliability of this analysis, data were routinely analyzed by two investigators independently.

### PIP_2_ staining

5C.C7 T cells expressing different GFP-tagged PIP5K isoforms were FACS sorted for matching numbers of GFP positive and negative T cells, using the same sort windows as in all other experiments. Both populations were mixed, adhered to a poly d-lysine-coated cover slip, and stained for PIP_2_ as established [28]. Briefly, cells were fixed in 4% Paraformaldehyde, permeabilized with 0.5% Saponin, and stained with an anti-PIP_2_ antiserum and an anti-mouse antibody conjugated to Alexa 568. Signal was >20-fold above non-specific staining background. Three-dimensional images of the stained cells were acquired and 100–200 cells per experiment were analyzed for PIP_2_ staining intensity, separately in the same field for GFP positive and negative cells: T cells were identified by intensity thresholding and the integrated Alexa 568 fluorescence was measured as a readout of total cellular PIP_2_ amounts.

### Biochemical and functional assays

To determine PIPI5K mRNA levels, cDNA from primary 5C.C7 T cells was analyzed by real time PCR using SYBR Green labeling and the Applied Biosystems 7300 real time PCR system with β2-microglobulin as the quantification standard. cDNA was prepared using the RNA STAT-60 reagent (Tel Test, Inc.) and SuperScript reverse transcriptase (Invitrogen) according to manufacturer's instructions. Phosphorylation of LAT and PLCγ was determined by Western blotting of cell extracts from T cell/APC couples, as described [6]. The phosphorylation of ERM proteins was similarly determined after T cell stimulation for 2 min with 10 µg/ml α-CD3 and α-CD28 and secondary antibody crosslinking. ERM and PIP5K γ expression were determined similarly in cell extracts from non-stimulated cells. Staining with Alexa 594 phalloidin was performed according to the instructions provided by the manufacturer. IL-2 was measured in T cell/APC culture supernatants after 16 h of cell contact using the OptEIA kit from DB Biosciences according to the instructions provided by the manufacturer as scaled down to as few as 10,000 sorted T cells.

## Supporting Information

Figure S1 **Different PIP5K isoforms display distinct spatiotemporal patterns.** **A.** The mRNA abundance of different PIP5K isoforms in 5C.C7 T cells was determined by real time PCR with β2 microglobulin mRNA as a standard and is given relative to the amount of β2 microglobulin mRNA with standard errors as indicated. Averages of 5–6 independent experiments are given. PIP5K β mRNA could be detected only once. **B–E.** Representative interactions of 5C.C7 T cells transduced with PIP5K γ87-GFP (B), α-GFP (C), β-GFP (D), and γ90-GFP (E) with CH27 APCs in the presence of 10 µM MCC peptide are shown at the indicated time points (in minutes) relative to the time of tight cell coupling. Differential interference contrast (DIC) images are shown on top, with top-down, maximum projections of 3-dimensional GFP fluorescence data at the bottom. GFP fluorescence intensity is displayed in a rainbow-like false-color scale (increasing from blue to red). Movies covering the entire time frames are in [6](for PIP5K γ87) and as Movies S1, S2, S3.
(EPS)

Figure S2 **PIP5K overexpression alters PIP_2_ levels and localization.** **A, B.** To determine spatiotemporal distributions of PIP5K-GFP isoforms, 5C.C7 T cells transduced to express a PIP5K-GFP isoform were FACS-sorted into a 5-fold range of expression centered at 3±0.6 µM that is minimally required for detection by fluorescence microscopy [6]. (A) To determine such PIP5K-GFP expression relative to endogenous PIP5K, cell extracts from 5C.C7 T cells expressing GFP fusions with PIP5K γ 87 or γ 90 and from matching numbers of non-transduced control cells were blotted for both PIP5K γ isoforms (on top) or for PIP5K γ90 only (on the bottom). Representative blots are shown. A suitable antibody against the β isoform is not available. (B) Based on previous calibration of GFP fluorescence intensity as a function of GFP expression [6](experimental procedures) endogenous PIP5K γ expression was calculated using the ratio of band intensities for PIP5K γ-GFP and PIP5K γ and is given with standard errors based on at least 2 independent experiments. **C.** In many experiments throughout this manuscript non-fluorescent PIP5K isoforms were overexpressed alongside imaging sensors using the same retroviral vector backbone used for the expression of the GFP-tagged PIP5K isoforms only. T cells expressing non-fluorescent PIP5K and a sensor where then sorted for low sensor expression, as established [6]. This resulted in expression levels of overexpressed non-fluorescent PIP5K similar to that of PIP5K-GFP: Cell extracts from 5C.C7 T cells expressing PIP5K γ87-GFP or PIP5K γ87 together with TCRζ-GFP and from matching numbers of non-transduced control cells were blotted for both PIP5K γ isoforms. One representative blot is shown. **D.** A representative PIP_2_ staining experiment is shown. FACS-sorted T cells expressing PIP5K β-GFP and non-transduced control cells were mixed in equal numbers, fixed, and stained with anti-PIP_2_ antiserum followed by an Alexa 568-conjugated secondary antibody. A series of matching bright field, GFP, and Alexa 568 images is shown. **E.** A representative interaction of a PLCδ PH-GFP-transduced 5C.C7 T cell with a CH27 APC in the presence of 10 µM MCC peptide is shown as in Fig. S1B. A movie covering the entire time frame is in [6]. **F–H.** Similar to E, the panels show representative interactions for 5C.C7 T cells expressing PLCδ PH-GFP with concurrent overexpression of PIP5K β (F), PIP5K γ90 (G), or PIP5K γ87 (H). Movies covering the entire time frame are given as Movies S4, S5, S6. **I.** Similar to Fig. 2B, the panel shows patterning data for 5C.C7 T cells expressing PLCδ PH-GFP with concurrent overexpression of PIP5K γ87. Given is the percentage accumulation of PLCδ PH-GFP with standard errors with the indicated patterns [6] relative to tight cell coupling. 29 cell couples were analyzed. **J.** For shRNA-mediated knockdown of the PIP5K γ isoforms, we used a retrovirally-expressed hairpin that targets both the γ87 and the γ90 isoforms. shRNA-mediated knockdown reduced PIP5K γ90 expression by 56±21% (p = 0.05). A representative Western blot is shown. Knockdown of combined PIP5K γ87/γ90 was less, not reaching significance any more. PIP_2_ levels as determined by immunofluorescence similar to Fig. 2A were reduced by about 5% (not reaching statistical significance), consistent with tight regulation of PIP_2_ levels. **K.** To block PIP_2_, PLCδ PH was employed as an E. coli expressed protein transduction reagent (‘tatPLCδ PH’). Effects of tatPLCδ PH on T cell activation were highly dose-dependent, consistent with tight regulation of PIP_2_ effector functions. While at 1 µM tatPLCδ PH substantial effects could not be found, at >5 µM tatPLCδ PH T cell coupling upon APC contact was reduced by >65% (p<0.001) from 50±5% to less than 20%. This was most likely caused by a>95% decrease (p<0.001) in the percentage of T cells with overt migratory polarity. We therefore used tatPLCδ PH at 5 µM, the maximal concentration allowing effective cell coupling. To directly assess the efficacy of 5 µM tat PLCδ PH, we tested its ability to compete with PLCδ PH-GFP FACS-sorted to a concentration of 2 µM. Similar to Fig. 2B, the panel shows patterning data for 5C.C7 T cells expressing PLCδ PH-GFP with concurrent PIP_2_ blockade by T cell pretreatment with 5 µM tat PLCδ PH. Given is the percentage of cell couples with standard errors that displayed accumulation of PLCδ PH-GFP with the indicated patterns [6] relative to tight cell coupling. Only 14 cell couples could be analyzed. Preincubation of T cells with 5 µM tatPLCδ PH made interface PLCδ PH-GFP accumulation moderately but significantly more transient with reduced accumulation at the time of tight cell couple formation (83±5% to 50±13%, p<0.005) and 80–120 s thereafter (60±4% to 43±8%, p = 0.05), thus defining the efficacy of tatPLCδ PH.
(EPS)

Figure S3 **Manipulation of PIP5K expression affects IL-2 secretion.** To determine whether reduced PIP_2_ generation would interfere with T cell activation at limiting activation conditions, 5C.C7 T cells were activated by CH27 APCs in the presence of 0.1–10 µM MCC peptide as indicated for 16 h upon knockdown of PIP5K γ. Cell culture supernatants were analyzed for IL-2 by ELISA and data are displayed similar to Fig. 3B. Data from 3 independent experiments are given.
(EPS)

Figure S4 **PIP5K overexpression rigidifies T cells.** **A.** 5C.C7 T cells transduced with TCRζ-GFP and PIP5K γ87 were activated with CH27 APCs and 10 µM MCC agonist peptide. Two interactions of such T cells binding to the same APC at the center of the image with no (top) or a very small (bottom) visible lamellum are shown as in Fig. S1B. The time of tight cell coupling could only be guessed. A movie covering the entire time frame is given as Movie S7. **B.** To distinguish between effects of PIP5K β and γ90 in uropod retraction, we counteracted PIP5K overexpression by parallel expression of PLCδ PH-GFP. Only PIP5K β-overexpressing T cells still showed delayed uropod retraction: 5C.C7 T cells transduced with PLCδ PH-GFP were activated with CH27 APCs and 10 µM MCC agonist peptide. The percentage of cell couples with a visible uropod is given with standard errors relative to tight cell coupling upon manipulation of PIP5K expression, as indicated. 19–74 cell couples were analyzed per condition. The most severe effect of PIP5K β overexpression on uropod retraction is consistent with its exclusive distal localization (Fig. 1C). **C, F, I, K.** As T cell spreading (Fig. 4A) is actin-driven, we assessed changes in T cell actin dynamics upon manipulation of PIP_2_ generation and access. Representative interactions of actin-GFP-transduced 5C.C7 T cells with CH27 APCs in the presence of 10 µM MCC peptide are shown as in Fig. S1B for a control (C), upon overexpression of PIP5K β (F), upon PIP5K γ knockdown (I), or upon T cell pretreatment with 5 µM tat PLCδ PH (K). Movies covering the entire time frame are given as Movies S8, S9, S10, S11. **D, E, G, H, J, L.** 5C.C7 T cells were transduced to express actin-GFP and activated with CH27 APCs and 10 µM MCC agonist peptide. The graphs show the percentage of cell couples with standard errors that displayed accumulation of actin-GFP with the indicated patterns [6] relative to tight cell coupling upon manipulation of the expression of PIP5K and PIP_2_ blockade as indicated. 29–60 cell couples were analyzed per condition. In non-transduced control T cells, actin rapidly and transiently spread to the periphery of the T cell/APC interface (C, D). At the time of tight cell couple formation 68±6% of cell couples displayed peripheral actin accumulation (D). The percentage of cell couples with peripheral accumulation rapidly declined to 13±5% at 3 min. Overexpression of PIP5K β, γ87, or γ90 all interfered with actin spreading to the interface periphery (E–H). The frequency of peripheral accumulation was reduced with a concomitant increase in diffuse patterning. For example upon overexpression of PIP5K γ90, at the time of tight cell coupling the percentage of cell couples with peripheral actin-GFP accumulation was reduced from 68±6% to 38±6% (p<0.01), whereas diffuse accumulation was increased from 16±5% to 35±6% (p = 0.01)(D, G). Such differences were significant (p<0.05) at most time points between tight cell coupling and 2 min thereafter in the comparison of control T cells with T cell overexpressing each of the PIP5K isoforms. Knockdown of PIP5K γ promoted peripheral actin accumulation by making it more sustained (I, J). From 1 to 5 min after tight cell coupling the percentage of cell couples with peripheral actin accumulation was significantly (p<0.05) enhanced at each time point in the knockdown T cells compared to control. Upon blocking access to PIP_2_ with 5 µM tatPLCδ PH (K, L), actin dynamics were largely unaltered, consistent with intact T cell spreading (Fig. 4A).
(EPS)

Figure S5 **PIP5K overexpression does not impair ERM expression, T cell coupling, or F-actin amounts.** **A.** To assess whether manipulation of PIP5K expression altered ERM expression, 5C.C7 T cell extracts were blotted for ERM proteins. ERM expression with standard errors upon overexpression/knockdown of the indicated PIP5K isoforms is given relative to non-transduced control cells. **B.** T cell spreading is a TCR-dependent process. Therefore, impaired T cell spreading could be the consequence of impaired TCR engagement. The ability of a T cell to form a cell couple upon initial APC contact is the most immediate readout of TCR engagement upon APC contact, occurring in seconds. The percentage of 5C.C7 T cells that form a tight cell couple upon contact with CH27 APCs in the presence of 10 µM MCC peptide is given with standard errors upon overexpression of PIP5K as indicated. 61–101 cell couples were analyzed per condition. An investigation of the modest differences in cell coupling upon PIP5K overexpression is beyond the scope of this study. However, because cell coupling is not reduced, it can be safely concluded that TCR engagement was not impaired. **C.** T cell rigidification could be caused by altered cellular F-actin amounts. We therefore determined F-actin amounts by phalloidin staining prior and past TCR engagement. T cell activation was mediated by antibodies, as the determination of T cell F-actin amounts in T cell/APC couples, where both cell types contain F-actin, is challenging. 5C.C7 T cells were activated with α-CD3 and α-CD28 antibodies for 2 min upon manipulation of PIP5K expression and PIP_2_ blockade as indicated. F-actin contents were determined by FACS analysis of Phalloidin-stained T cells. Mean intensity of Phalloidin staining is displayed with standard errors relative to that of non-transduced, non-stimulated T cells. Data from three independent experiments are given. Consistent with actin-driven T cell spreading upon APC contact, T cell stimulation with α-CD3 and α-CD28 triggered a significant (p<0.001) increase in T cell F-actin contents in control cells by 23±3%. PIP5K overexpression and knockdown did not substantially alter T cell F-actin amounts relative to control neither prior nor post T cell stimulation. PIP_2_ blockade modestly increased F-actin amounts prior to T cell activation (p<0.05 as indicated with an asterisk) but not thereafter. An investigation of this effect is beyond the scope of this study.
(EPS)

Figure S6 **PIP_2_ manipulation interferes with accumulation of the TCR at the center of the T cell/APC interface.** **A.** A representative interaction of a TCRζ-GFP-transduced 5C.C7 T cell with a CH27 APC in the presence of 10 µM MCC peptide is shown at the indicated time points (in minutes) relative to the time of tight cell coupling as in Fig. S1B. A movie covering the entire time frame is given as Movie S12. **B–I.** Representative images and patterning data for 5C.C7 T cells expressing TCRζ-GFP upon concurrent overexpression of PIP5K γ90 (B), β (C), γ87 (D, E), a knockdown cassette for PIP5K γ (F, G), or T cell treatment with 5 µM tat PLCδ PH (H, I) are displayed as in Fig. S1B (images) or Fig. 6A (patterning data). Movies covering the entire time frame are given as Movies S13, S14, S15, S16, S17. 32–56 cell couples were analyzed per condition.
(EPS)

Table S1 PIP2 controls T cell activation by regulating T cell rigidity and spatiotemporal organization.
(DOC)

Movie S1 A representative interaction of sensor-transduced 5C.C7 T cell with a CH27 B cell lymphoma APC in the presence of 10 µM MCC agonist peptide is shown in this and all subsequent movies. Movies were acquired using time-lapse epifluorescence microscopy. Differential interference contrast (DIC) images are shown on top, with matching top-down, maximum projections of 3-dimensional sensor fluorescence data on the bottom. The sensor fluorescence intensity is displayed in a rainbow-like false-color scale (increasing from blue to red). 20 s intervals in movie acquisition are played back as 2 frames per second. The individual captions list for each movie the figure it refers to, the sensor and any non-fluorescent additional proteins expressed by the 5C.C7 T cell, the time of T cell/APC couple formation given both as the movie frame number and the displayed movie time (in parentheses), and noteworthy features of the movie. Movie S1 refers to Fig. S1C, the 5C.C7 T cells are transduced with PIP5K α-GFP, cell coupling occurs in frame 3 (1s), and prominent are a large lamellum upon cell coupling and transient interface accumulation of PIP5K α-GFP.
(MOV)

Movie S2 Refers to Fig. S1D, the 5C.C7 T cells are transduced with PIP5K β-GFP, cell coupling occurs in frame 7 (3s), and prominent are a small lamellum upon cell coupling and sustained distal accumulation of PIP5K β-GFP.
(MOV)

Movie S3 Refers to Fig. S1E, the 5C.C7 T cells are transduced with PIP5K γ90-GFP, cell coupling occurs in frame 5 (2s), and prominent are a small lamellum upon cell coupling and initial distal accumulation of PIP5K γ90-GFP followed by late interface accumulation.
(MOV)

Movie S4 Refers to Fig. S2H, the 5C.C7 T cells are transduced with PLCδ PH-GFP and PIP5K γ87, cell coupling occurs in frame 9 (4s), and prominent is transient lamellal accumulation of PLCδ PH-GFP. The T cell transits to an adjacent APC during the movie. Only data from the 1^st^ T cell/APC interaction are included in the analysis.
(MOV)

Movie S5 Refers to Fig. S2G, the 5C.C7 T cells are transduced with PLCδ PH-GFP and PIP5K γ90, cell coupling occurs in frame 5 (2s), and prominent is transient interface accumulation of PLCδ PH-GFP. It is better sustained than that in the control T cell/APC interactions.
(MOV)

Movie S6 Refers to Fig. S2F, the 5C.C7 T cells are transduced with PLCδ PH-GFP and PIP5K β, cell coupling occurs in frame 2 (0s), and prominent is sustained distal accumulation of PLCδ PH-GFP.
(MOV)

Movie S7 Contains two T cell/APC interactions, as indicated by the positions of the respective T cells relative to the APC. Movie S7 refers to Fig. S4A, the 5C.C7 T cells are transduced with TCRξ-GFP and PIP5K γ87, cell coupling cannot be timed precisely, and prominent is that in particular for the T cell on top a lamellum mediating cell coupling cannot be detected.
(MOV)

Movie S8 Refers to Fig. S4C, the 5C.C7 T cells are transduced with actin-GFP, cell coupling occurs in frame 2 (0s), and prominent is initial peripheral actin-GFP interface accumulation followed by variable patterns with diminishing intensity.
(MOV)

Movie S9 Refers to Fig. S4F, the 5C.C7 T cells are transduced with actin-GFP and PIP5K β, cell coupling occurs in frame 6 (2s), and prominent is transient diffuse actin-GFP interface accumulation.
(MOV)

Movie S10 Refers to Fig. S4I, the 5C.C7 T cells are transduced with actin-GFP and sh PIP5K γ, cell coupling occurs in frame 4 (1s), and prominent is early peripheral actin-GFP interface accumulation that is sustained in various patterns.
(MOV)

Movie S11 Refers to Fig. S4K, the 5C.C7 T cells are transduced with actin-GFP and treated with 5 µM tat PLCδ PH, cell coupling occurs in frame 3 (1s), and prominent is transient lamellal and diffuse actin-GFP interface accumulation.
(MOV)

Movie S12 Refers to Fig. S6A, the 5C.C7 T cells are transduced with TCRξ-GFP, cell coupling occurs in frame 7 (3s), and prominent is rapid and sustained TCRξ-GFP accumulation at the center of the T cell/APC interface.
(MOV)

Movie S13 Refers to Fig. S6B, the 5C.C7 T cells are transduced with TCRξ-GFP and PIP5K γ90, cell coupling occurs in frame 6 (2s), and prominent is initial distal with delayed central TCRξ-GFP accumulation.
(MOV)

Movie S14 Refers to Fig. S6C, the 5C.C7 T cells are transduced with TCRξ-GFP and PIP5K β, cell coupling occurs in frame 5 (2s), and prominent are delayed weak central TCRξ-GFP interface accumulation and subsequent removal of TCRζ-GFP from the interface with accumulation in internal structures, likely vesicles.
(MOV)

Movie S15 Refers to Fig. S6D, the 5C.C7 T cells are transduced with TCRξ-GFP and PIP5K γ87, cell coupling occurs in frame 4 (1s), and prominent is delayed accumulation of TCRξ-GFP at the center of the T cell/APC interface.
(MOV)

Movie S16 Refers to Fig. S6F, the 5C.C7 T cells are transduced with TCRξ-GFP and sh PIP5K γ, cell coupling occurs in frame 3 (1s), and prominent is transient accumulation of TCRξ-GFP at the center of the T cell/APC interface.
(MOV)

Movie S17 Refers to Fig. S6H, the 5C.C7 T cells are transduced with TCRξ-GFP and treated with 5 µM tat PLCδ PH, cell coupling occurs in frame 7 (3s), and prominent is sustained central TCRξ-GFP interface accumulation without initial distal accumulation.
(MOV)
