# Supplementary material for: Phosphatidylinositol (4,5) Bisphosphate Controls T Cell Activation by Regulating T Cell Rigidity and Organization
Source: PLoS One. 2011 Nov 11;6(11):e27227. doi: 10.1371/journal.pone.0027227 (PMC3214035; doi:10.1371/journal.pone.0027227)
Supplement: Table S1 — PIP2 controls T cell activation by regulating T cell rigidity and spatiotemporal organization. (DOC) [file pone.0027227.s007.doc]

| **Table S1 PIP_2_ controls T cell activation by regulating T cell rigidity and spatiotemporal organization** | | | | | | | | |
| --- | --- | --- | --- | --- | --- | --- | --- | --- |
| **Element of T cell function** | **PIP5K overexpression** | | | | **Order of severity** | **γ shRNA** | **Tat PLCδ PH** | **Conclusion** |
|  | **PIP5K β** | **PIP5K γ90** | **PIP5K γ87** | |  |  |  |  |
| PIP_2_ levels | enhanced by 20 % | | | | β=γ90=γ87 | small decrease not reaching significance | N/A | PIP5K overexpression yields increased PIP_2_ levels |
| Distribution of accessible PIP_2_ | Redirected from the interface to the distal pole | More sustained interface accumul. | Marginal changes only | | β>γ90>γ87 | N/A | Less accessible PIP_2_ without pattern changes | Local changes in PIP_2_ generation yield localized changes in accessible PIP_2_ |
| Interface diameter | Decreased upon cell coupling | | | | β=γ90=γ87 | Not changed | | Globally enhanced PIP_2_ generation rigidifies the T cell at the T cell/APC interface, likely through inhibition of ERM de-phosphorylation |
| Cell coupling without lamellum | Increased | | | | β=γ90=γ87 | Not changed | |  |
| ERM phosphorylation | n.d. | Inhibition of ERM dephosphorylation upon T cell activation | | | γ90=γ87 | Not changed | n.d. |  |
| Uropod retraction | Delayed | | Not changed | | β>γ90 | Not changed | | T cell rigidity at the distal pole and TCR localization are regulated by distal PIP_2_ |
| Interface TCR clustering | Delayed | | | | β,γ90>γ87 | Not changed | Reduced |  |
| Distal TCR accumulation | Inhibited recruitment, delayed release | Delayed release | | | β>γ90,γ87 | Not changed | Inhibited recruitment |  |
| Proximal T cell signaling | n.d. | Inhibited | | Not significantly changed | γ90>γ87 | Enhanced | n.d. | Enhanced PIP_2_ in particular at the distal pole, inhibits T cell activation |
| IL-2 secretion | Inhibited | | | Not significantly changed | β>γ90>γ87 | Modestly enhanced | |  |
